# Supplementary material for: Kinetic analysis of ASIC1a delineates conformational signaling from proton-sensing domains to the channel gate
Source: eLife. 2021 Mar 17;10:e66488. doi: 10.7554/eLife.66488 (PMC8009679; doi:10.7554/eLife.66488)
Supplement: Supplementary file 1. — The decay kinetics were determined as decay time, the time to pass from 90% to 10% of the maximal amplitude (RTFoff). Note that for mutants whose signal was not sustained, the RTFoff could not be determined. [file elife-66488-supp1.docx]

| Kinetic analysis of ASIC1a delineates conformational signaling from proton-sensing domains to the channel gate |
| --- |
| *Sabrina Vullo, Nicolas Ambrosio, Jan P. Kucera, Olivier Bignucolo and Stephan Kellenberger* |

**Supplementary File 1. Decay kinetics of the ΔF signal when switching back from pH6.0 to pH7.4**

| **Mutant** | **Decay time (ms)** | | | **n** |
| --- | --- | --- | --- | --- |
| E63C | 224 | ± | 61 | 4 |
| Y71C | 2083 | ± | 81 | 9 |
| H72C | 2839 | ± | 485 | 7 |
| K424C | 347 | ± | 40 | 13 |
| A425C | 2923 | ± | 145 | 10 |
| I428C | 4134 | ± | 348 | 10 |
|  |  |  |  |  |
| K105C | 1898 | ± | 142 | 9 |
| K133C | 3311 | ± | 241 | 12 |
| I137C | 2702 | ± | 186 | 9 |
| E235C | 1916 | ± | 230 | 6 |
| E355C | 4033 | ± | 314 | 4 |
| K388C | 3679 | ± | 224 | 7 |
| Y389C | 2555 | ± | 258 | 7 |
| K392C | 5529 | ± | 681 | 8 |
|  |  |  |  |  |
| A81C | 851 | ± | 123 | 5 |
| S83C | 9329 | ± | 640 | 7 |
| Q84C | 3569 | ± | 649 | 7 |
|  |  |  |  |  |
| A81C Y417V P205W | 290 | ± | 15 | 5 |
| S83C Y417V P205W | 789 | ± | 136 | 6 |
| Q84C Y417V P205W | 767 | ± | 69 | 5 |
| Q84C Y417V R206W | 1105 | ± | 81 | 8 |
| Q84C Y417V L207W | 424 | ± | 33 | 10 |
| A81C Y417V K208W | 183 | ± | 16 | 6 |
| A81C Y418V T209W | 1024 | ± | 355 | 4 |
| A81C Y417V M210W | 1108 | ± | 109 | 6 |
|  |  |  |  |  |
| A81C Y417V T289W | 2162 | ± | 146 | 6 |
| S83C Y417V T289W | 5906 | ± | 575 | 5 |
| Q84C Y417V T289W | 3041 | ± | 117 | 5 |
| S83C Y417V D357W | 756 | ± | 53 | 5 |
| S83C Y417V Q358W | 362 | ± | 60 | 7 |
| S83C Y417V E359W | 1677 | ± | 129 | 5 |
| A81C Y417V L369W | 347 | ± | 37 | 5 |
| S83C Y417V L369W | 165 | ± | 47 | 3 |
