# Supplementary material for: Kinetic analysis of ASIC1a delineates conformational signaling from proton-sensing domains to the channel gate
Source: eLife. 2021 Mar 17;10:e66488. doi: 10.7554/eLife.66488 (PMC8009679; doi:10.7554/eLife.66488)
Supplement: Supplementary file 2. — The modeling of the ΔF traces is described in Materials and methods. Note that F(C) was always = 0, except for S83C Y417V D357W where it was set to −0.15. a, F(OD) calculated from Fsust/Fpeak ratio; b, parameters chosen to reproduce the shape of the trace; c, F(O) calculated from the fluorescence rise time (RTF); d, F(O) and F(OD) determined from the amplitude ratio of the two components of the ΔF signal; *, experimental traces not matched by model, off kinetics are too slow in the model; & , F(C)=−0.15. F(CD) was in all cases except for b calculated from the RTF when the pH was switched back to pH7.4. [file elife-66488-supp2.docx]

| Kinetic analysis of ASIC1a delineates conformational signaling from proton-sensing domains to the channel gate | | | |
| --- | --- | --- | --- |
| *Sabrina Vullo, Nicolas Ambrosio, Jan P. Kucera, Olivier Bignucolo and Stephan Kellenberger* | | | |
|  |  |  |  |
| **Supplementary File 2. Parameters of kinetic models for the simulation of ΔF traces** | | | |

| **Mutant** | **F(O)** | **F(OD)** | **F(CD)** | **comment** |
| --- | --- | --- | --- | --- |
| E63C | 1 | 0.16 | 0.01 | a |
| H70C | -1 | 0.1 | 0.1 | b |
| Y71C | -1 | -0.4 | -0.5 | b |
| H72C | -1 | -0.05 | -0.01 | a |
| T419C | -1 | 0 | 0 | b |
| K424C | -1 | -0.48 | -0.04 | a |
| A425C | -1 | -1 | -0.23 | c |
| I428C | -0.94 | -1 | -0.33 | c |
| K105C | 0.99 | 1 | 0.16 | c |
| K133C | 0.96 | 1 | 0.26 | c |
| I137C | 0.90 | 1 | 0.22 | c |
| E235C | 0.89 | 1 | 0.17 | c |
| E355C | -0.70 | 1 | 0.33 | d |
| K388C | 0.90 | 1 | 0.29 | c |
| Y389C | 0.90 | 1 | 0.21 | c |
| K392C | 0.88 | 1 | 0.50 | c |
|  |  |  |  |  |
| A81C | -0.86 | -1 | -0.11 | c |
| S83C | -0.77 | -1 | -1.00 | c |
| Q84C | -0.60 | -1 | -0.28 | c |
|  |  |  |  |  |
| A81C Y417V P205W | 0.83 | 1 | 0.08 | c |
| S83C Y417V P205W | -0.74 | -1 | -0.10 | c |
| Q84C Y417V P205W | -0.50 | 0.5 | -1.00 | b, d* |
| Q84C Y417V R206W | -0.49 | -1 | -0.12 | c |
| A81C Y417V L207W | -0.15 | 0 | -0.10 | b* |
| Q84C Y417V L207W | -0.85 | -1 | -0.08 | c |
| A81C Y417V K208W | -0.88 | -1 | -0.07 | c |
| A81C Y417V T209W | -1.00 | -1 | -0.11 | c |
| A81C Y417V M210W | 0.95 | 1 | 0.12 | c |
|  |  |  |  |  |
| A81C Y417V T289W | -0.85 | -1 | -0.18 | c |
| S83C Y417V T289W | -1.00 | -1 | -0.56 | c |
| Q84C Y417V T289W | -0.79 | -1 | -0.24 | c |
| S83C Y417V D357W | -0.60 | 0 | -0.30 | b*, & |
| S83C Y417V Q358W | 0.35 | -0.5 | -0.04 | b |
| S83C Y417V E359W | -0.93 | -1 | -0.15 | c |
| A81C Y417V L369W | -1.00 | 0.6 | 0.05 | b, d |
| S83C Y417V L369W | -0.70 | 1 | 0.07 | b, d |
