# Supplementary material for: Kinetic analysis of ASIC1a delineates conformational signaling from proton-sensing domains to the channel gate
Source: eLife. 2021 Mar 17;10:e66488. doi: 10.7554/eLife.66488 (PMC8009679; doi:10.7554/eLife.66488)
Supplement: Supplementary file 3. — The ratio of the ΔF/F of the acidification from the desensitized state (i.e., conditioning pH6.7, stimulation pH6)/ ΔF/F of the acidification from the closed state (pH7.4 / pH6) is shown as a percentage (n = 3–10, mean ± SEM). This ratio is not indicated here for the mutants of Figure 1 that had previously been measured with a different perfusion system. For those mutants, the ratio was <20% (Bonifacio et al., 2014; Gwiazda et al., 2015). For most mutants, the ratio was measured using the two fluorophores Alexa Fluor 488 and CF488. The fluorophore yielding the lower ratio was subsequently used for the experiments and its ratio (as a percentage) is indicated in this table. Negative values indicate that the two protocols produced signals of opposite polarity. Mutants labeled by CF488 are marked with C. Alexa Fluor 488 was used for all the remaining mutants. [file elife-66488-supp3.docx]

Kinetic analysis of ASIC1a delineates conformational signaling from proton-sensing domains to the channel gate

## *Sabrina Vullo, Nicolas Ambrosio, Jan P. Kucera, Olivier Bignucolo and Stephan Kellenberger*

## **Supplementary File 3. Test for intrinsic pH dependence of fluorophores**

| Mutant | Ratio (%) ΔF/F acidification from desensitized/ ΔF/F acidification from closed | |
| --- | --- | --- |
| *Wrist mutants* | |  |
| E63C^C^ | | 10 ± 11 |
| H70C | | -4 ± 3 |
| Y71C | | -19 ± 3 |
| H72C | | 6 ± 3 |
| T419C | | 33 ± 7 |
| K424C | | 15 ± 3 |
| A425C | | 13 ± 7 |
| I428C | | 23 ± 4 |
|  | |  |
| Palm single mutants | |  |
| A81C | | -2 ± 2 |
| S83C | | 1 ± 1 |
| Q84C | | 10 ± 3 |
|  | |  |
| Palm - intrasubunit | |  |
| A81C Y417V P205W | | -2 ± 3 |
| S83C Y417V P205W | | 10 ± 3 |
| Q84C Y417V P205W 1^st^ signal (1^st^ peak) | | 7 ± 22 |
| Q84C Y417V P205W 2^nd^ signal | | 199 ± 43 |
| Q84C Y417V P205W 2^nd^ peak | | 0 ± 4 |
| Q84C Y417V R206W | | 16 ± 2 |
| A81C Y417V L207W 1^st^ signal | | 0 ± 2 |
| A81C Y417V L207W 2^nd^ signal | | 123 ± 50 |
| Q84C Y417V L207W | | 26 ± 10 |
| A81C Y417V K208W | | 13 ± 18 |
| A81C Y417V T209W | | -1 ± 1 |
| A81C Y417V M210W | | 32 ± 13 |
|  | |  |
| Palm - intersubunit | |  |
| A81C Y417V T289W | | 5 ± 1 |
| S83C Y417V T289W | | 10 ± 3 |
| Q84C Y417V T289W | | 22 ± 2 |
| S83C Y417V D357W 1^st^ signal (1^st^ peak) | | 19 ± 4 |
| S83C Y417V D357W 2^nd^ signal | | 357 ± 110 |
| S83C Y417V D357W 2^nd^ peak | | 10 ± 2 |
| S83C Y417V Q358W 1^st^ signal | | 35 ± 34 |
| S83C Y417V Q358W 2nd signal | | -3 ± 30 |
| S83C Y417V E359W | | 44 ± 20 |
| A81C Y417V L369W 1^st^ signal | | -34 ± 5 |
| A81C Y417V L369W 2^nd^ signal | | 28 ± 9 |
| S83C Y417V L369W 1^st^ signal | | 73 ± 18 |
| S83C Y417V L369W 2^nd^ signal | | -33 ± 29 |
