# Supplementary material for: Kinetic analysis of ASIC1a delineates conformational signaling from proton-sensing domains to the channel gate
Source: eLife. 2021 Mar 17;10:e66488. doi: 10.7554/eLife.66488 (PMC8009679; doi:10.7554/eLife.66488)
Supplement: Supplementary file 4. — This table indicates the sequences of the oligonucleotides used for mutagenesis. [file elife-66488-supp4.docx]

Kinetic analysis of ASIC1a delineates conformational signaling from proton-sensing domains to the channel gate

## *Sabrina Vullo, Nicolas Ambrosio, Jan P. Kucera, Olivier Bignucolo and Stephan Kellenberger*

## **Supplementary File 4. Sequences of oligonucleotides for mutagenesis**

| **Mutation** | **Forward oligonucleotide** | **Reverse oligonucleotide** |
| --- | --- | --- |
| E63C | CTGTGTGTGTGCACGTGCCGTGTGCAGTACTAC | GTAGTACTGCACACGGCACGTGCACACACACAG |
| H70C | CGTGTGCAGTACTACTTCTGTTACCACCATGTCACCAAG | CTTGGTGACATGGTGGTAACAGAAGTAGTACTGCACACG |
| Y71C | GCAGTACTACTTCCACTGTCACCATGTCACCAAGC | GCTTGGTGACATGGTGACAGTGGAAGTAGTACTGC |
| H72C | CAGTACTACTTCCACTACTGTCATGTCACCAAGCTCGAC | GTCGAGCTTGGTGACATGACAGTAGTGGAAGTAGTACTG |
| T419C | GAAGTCCTCAACTATGAGTGCATTGAACAGAAGAAGGC | GCCTTCTTCTGTTCAATGCACTCATAGTTGAGGACTTC |
| K424C | GAGACCATTGAACAGAAGTGTGCCTATGAGATTGCAGGG | CCCTGCAATCTCATAGGCACACTTCTGTTCAATGGTCTC |
| A425C | CATTGAACAGAAGAAGTGCTATGAGATTGCAGGGC | GCCCTGCAATCTCATAGCACTTCTTCTGTTCAATG |
| I428C | GAAGGCCTATGAGTGTGCAGGGCTCCTG | CAGGAGCCCTGCACACTCATAGGCCTTC |
| A81C | CAAGCTCGACGAGGTGTGTGCCTCTCAGCTTAC | GTAAGCTGAGAGGCACACACCTCGTCGAGCTTG |
| S83C | GAGGTGGCTGCCTGTCAGCTTACCTTC | GAAGGTAAGCTGACAGGCAGCCACCTC |
| Q84C | GAGGTGGCTGCCTCTTGTCTTACCTTCCCTGCTG | CAGCAGGGAAGGTAAGACAAGAGGCAGCCACCTC |
| Y417V | CATTTTCTTTGAAGTCCTCAACGTCGAGACCATTGAACAGAAGAAG | CTTCTTCTGTTCAATGGTCTCGACGTTGAGGACTTCAAAGAAAATG |
| P205W | CCGAGATGGGCGGTGGCGGCTGAAGAC | GTCTTCAGCCGCCACCGCCCATCTCGG |
| R206W | GAGATGGGCGGCCGTGGCTGAAGACCATG | CATGGTCTTCAGCCACGGCCGCCCATCTC |
| L207W | GGGCGGCCGCGGTGGAAGACCATGAAG | CTTCATGGTCTTCCACCGCGGCCGCCC |
| K208W | GCGGCCGCGGCTGTGGACCATGAAGGATG | CATCCTTCATGGTCCACAGCCGCGGCCGC |
| T209W | CGGCCGCGGCTGAAGTGGATGAAGGATGGGAC | GTCCCATCCTTCATCCACTTCAGCCGCGGCCG |
| M210W | CGCGGCTGAAGACCTGGAAGGATGGGACG | CGTCCCATCCTTCCAGGTCTTCAGCCGCG |
| T289W | CCCCACCCTGGGGCTGGTGCAAAGCTGTTACC | GGTAACAGCTTTGCACCAGCCCCAGGGTGGGG |
| D357W | CTTCCTGGTGGAGAAGTGGCAGGAGTACTGCGTGTG | CACACGCAGTACTCCTGCCACTTCTCCACCAGGAAG |
| Q358W | CTGGTGGAGAAGGACTGGGAGTACTGCGTGTG | CACACGCAGTACTCCCAGTCCTTCTCCACCAG |
| E359W | GTGGAGAAGGACCAGTGGTACTGCGTGTGTG | CACACACGCAGTACCACTGGTCCTTCTCCAC |
| L369W | GTGAAATGCCTTGCAACTGGACCCGCTATGGCAAAG | CTTTGCCATAGCGGGTCCAGTTGCAAGGCATTTCAC |
